# Theoretical Study on the Metabolic Mechanism of Heptachlor in Human Cytochrome P450 Enzymes

**DOI:** 10.3390/ijms26052021

**Published:** 2025-02-26

**Authors:** Xuerui Zhao, Hao Zhang, Xiaoli Shen, Qingchuan Zheng, Song Wang

**Affiliations:** 1 Institute of Theoretical Chemistry, Jilin University, Changchun 130021, China; zhaoxr22@mails.jlu.edu.cn (X.Z.); stringbell@jlu.edu.cn (H.Z.); shenxl22@mails.jlu.edu.cn (X.S.); 2 School of Pharmaceutical Sciences, Jilin University, Changchun 130021, China; zhengqc@jlu.edu.cn

**Keywords:** heptachlor, cytochrome P450 enzyme, metabolic mechanism, MD simulation, QM calculation

## Abstract

Heptachlor (HEP) is an insecticide metabolized by cytochrome P450 (CYP) enzymes in the human liver, resulting in the formation of heptachlor epoxide (HEPX). HEPX can persist in the human body for a long duration. Therefore, it can be extremely harmful. A comprehensive understanding of HEP’s metabolic fate may provide a theoretical basis for mitigating associated hazards. However, the specific human CYP isoforms that metabolize HEP, and their metabolic mechanisms, remain unclear. In this study, eight human CYP isoforms were used as catalytic enzymes to investigate the metabolic mechanism of HEP using molecular docking, molecular dynamics simulations, and quantum mechanical calculations. These results indicate that HEP primarily binds to CYP enzymes through hydrophobic interactions, and that the binding positions of HEP are determined by the composition and shape of the hydrophobic pockets near the active site. Based on the reaction distance, CYP2A6, CYP3A4, and CYP3A5 were the only three enzymes that could metabolize HEP. The epoxidation of HEP catalyzed by the doublet state of compound I was effectively concerted, and the rate-determining step was the electrophilic attack of the oxygen atom on HEP. The energy barriers of the rate-determining step vary significantly among different enzymes. A comparison of these energy barriers suggested that CYP3A5 is the most likely enzyme for HEP catalysis in humans.

## 1. Introduction

**Figure 1:**
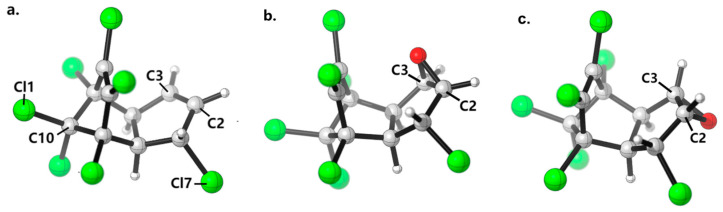
Schematic structures of HEP (**a**), endo-HEPX (**b**), and exo-HEPX (**c**), with C, O, Cl, and H being indicated in grey, red, green, and white, respectively.

The cyclopentadiene compound 1, 4, 5, 6, 7, 8, 8-c-3a, 4, 7, 7a-tetrahydro-4, 7-methylene-indene (C_10_Cl_7_H_5_), abbreviated as heptachlor (HEP, Figure 1a), was widely used as an insecticide against soil-borne insects and termites in developed countries during the 1960s and 1970s [1]. Studies have shown that HEP and its metabolites are potent carcinogens and neurotoxicants in living organisms, causing adverse effects on hormone secretion and the growth and development of human beings [2,3,4,5,6]. In 1984, HEP was classified by the World Health Organization as a Group 2B carcinogen [7]. However, because of its stable chemical structure, HEP can still be detected in the environment and organisms across many regions decades after its ban [8,9,10,11,12]. Studies on the metabolic mechanism of HEP may contribute to mitigating its toxic effects.

Previous studies have shown that HEP is initially absorbed through the gastrointestinal tract after ingestion by organisms such as rats, with it being metabolized in the liver to heptachlor epoxide (HEPX) within a short time [13,14,15,16]. Two isomers of HEPX exist, namely endo-HEPX and exo-HEPX, (Figure 1b,c), both of which are extremely persistent in the human body. Jonsson et al. detected HEPX in human blood, adipose tissue, and breast milk [17,18,19]. Adeshina et al. found that the concentration of HEPX in human adipose tissue samples increases with age [20]. Thus, HEPX could possibly be responsible for the long-term damage after HEP exposure in humans. Therefore, inhibiting the conversion of HEP to HEPX may help minimize HEP’s toxicity. Cytochrome P450 (CYP) enzymes are terminal monooxygenases of the mixed-function oxidase system belonging to the b family of cytochromes. The ability of CYP (cytochrome P450) to catalyze mono-oxygenation reactions is mainly attributed to a porphyrin π radical iron-based intermediate, which is called Compound I. Compound I is located in a large hydrophobic pocket at the active site. The distal end of the pocket is lined with key hydrophobic residues that can bind the substrate. These enzymes are widely distributed among plants, animals, and microorganisms, and they play an important role in the metabolism, activation, and degradation of endogenous substrates and exogenous compounds [21,22,23]. Studies have confirmed that CYPs play a crucial role in the epoxidation of HEP [24,25]. However, the CYP isoforms that metabolize HEP to HEPX and the mechanism of their interactions in humans are currently unknown. Further research is required to elucidate the mechanism underlying the toxic effects of HEP in humans.

In this study, we used molecular docking and molecular dynamics (MD) simulations to investigate the binding modes and key factors that influence the interactions between HEP and different CYP isoforms. The potential of these isoforms to catalyze HEP epoxidation was tentatively predicted. We then performed quantum mechanical (QM) calculations to identify the main reaction pathways for HEP epoxidation and further screen the key catalyzing enzymes. This study elucidated the interaction mechanism between HEP and human CYPs, filling the gap in the study of the microscopic metabolic mechanism of HEP in the human liver and providing a theoretical basis for studying the metabolic mechanism of other cyclopentadienyl pesticide residues in living organisms.

## 2. Results and Discussion

### 2.1. Different Binding Modes of HEP to Eight CYP Enzymes and Influencing Factors

**Figure 2:**
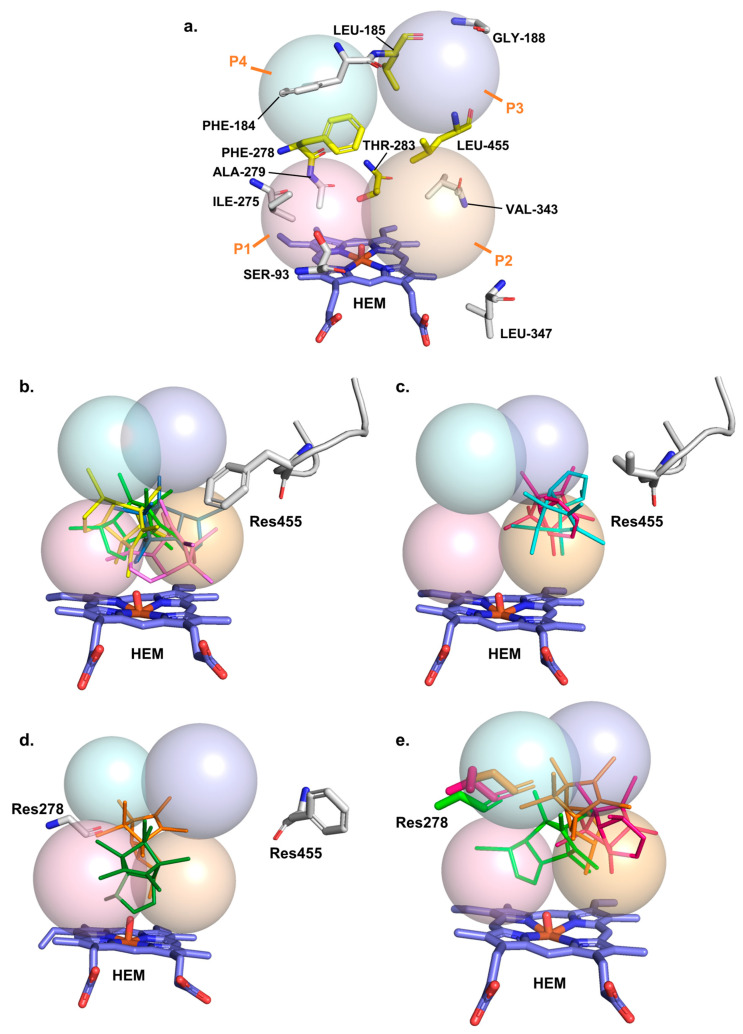
(**a**) Schematic representation of the four substrate-binding pockets in the crystal structure of CYP3A5, with the residues forming the pockets in white and yellow and the pockets-separating residues in yellow. In all the pictures, HEP is represented in a linear form, the residues are shown in a stick model, and Cpd I (without cysteine, i.e., HEM in the figure) is in slate blue. (**b**) The positions of HEP in CYP1A2-HEP (green), CYP2A6-HEP (yellow), CYP2E1-HEP (blue) and CYP3A5-HEP (violet). Res455 faces the P2 pocket, and HEP tends to dock into the P1/P2 pocket; (**c**) The positions of HEP in CYP2B6-HEP (cyan) and CYP2D6-HEP (hot pink). Res455 faces the P3 pocket, and HEP slightly shifts towards the P3 pocket; (**d**) The positions of HEP in CYP2C9-HEP (orange) and CYP3A4-HEP (forest green). Res455 points neither to the P2 nor to the P3, and the binding position of HEP is not affected by it, which is different from other complexes; (**e**) The positions of HEP in CYP1A2-HEP (green), CYP2C9-HEP (orange) and CYP2D6-HEP (hot pink). The side chain of Res278 is smaller than that in several other enzymes, and HEP occupies both pockets simultaneously.

To construct complex structures, we performed molecular docking and obtained 150 complex structures for each enzyme. The optimal complex conformations were screened based on the distance between the HEP and Compound I (Cpd I) and the binding energy. HEP is primarily docked in hydrophobic pockets near the active centers of enzymes, but the exact locations vary among different enzymes. We analyzed the influencing factors by comparing the conformations of the eight enzymes. Overlap analysis by Discovery Studio 3.0 showed that all eight proteins had initial crystal structure similarity scores of 0.5 or higher (out of 1.0), demonstrating the highly conserved spatial structures of these enzymes [21]. The binding pockets of the CYP enzymes with high homology are composed almost entirely of hydrophobic amino acid residues [26]. Consequently, we divided the ligand binding region into four hydrophobic pockets (P1–P4) for binding HEP. An example of the P1–P4 arrangement in CYP3A5 is shown in Figure 2a. Because the residue sequences at the corresponding positions in each of the seven proteins varied, we described the four hydrophobic pockets by Res + residue numbers in CYP3A5 (e.g., Res455), with the corresponding residues in the other enzymes listed in Table S1. According to the above rule, the P1 pocket consists of Res275, Res278, Res279, Res283, and Cpd I, with Res347 being a part of this pocket in some proteins. The P2 pocket consists of Res283, Res343, Res455, and Cpd I. Res347 also belongs to this pocket in certain proteins. HEP was positioned closer to Cpd I when bound to the P1/P2 pocket. The P3 pocket contains Res185 and Res188, whereas the P4 pocket contains Res184, Res185, and Res278. When HEP was bound to the P3/P4 pocket, it was farther from Cpd I. Res283, Res455, Res185, and Res278 serve as the residues that separate the four pockets, with their side chains oriented toward the center of the entire substrate-binding region.

Comparison of the conformations of the eight complexes showed that Res278 and Res455, which separated the P1 and P4 and P2 and P3 pockets, respectively, significantly influenced the initial docking positions of HEP. When Res455 was oriented toward the P2 pocket (as seen in CYP1A2-HEP, CYP2A6-HEP, CYP2E1-HEP, and CYP3A5-HEP), HEP tended to dock to the P1/P2 pocket (Figure 2b). When Res455 was oriented toward the P3 pocket (as seen in CYP2B6-HEP and CYP2D6-HEP), it no longer separated the P2 and P3 pockets. Consequently, the HEP shifted slightly away from the P2 pocket and moved a little toward the P3 pocket (Figure 2c). However, in CYP2C9-HEP and CYP3A4-HEP, Res455 was not directed toward P2 or P3 but toward the back of the binding pocket. The substrate was unaffected by its orientation, and the binding position of HEP was somewhat different from that of the other six complexes (Figure 2d: the binding position of HEP in CYP3A4-HEP appears similar to that in Figure 2b from this angle but is shifted more toward the outside of the paper). Although the position of Res278 in CYP1A2-HEP, CYP2C9-HEP, and CYP2D6-HEP was not very different from that in other enzymes, it was either glycine or serine, both of which have smaller side chains. Therefore, no clear demarcation was observed between the P1 and P4 pockets in these complexes, thereby allowing HEP to occupy both pockets simultaneously (Figure 2e). Notably, neither Res278 nor Res455 in CYP2C9-HEP served to separate the pockets, resulting in a more accommodating substrate-binding space in this protein where the substrate docked in the gap between the four pockets (Figure 2e).

### 2.2. Preliminary Prediction of CYP Enzymes That May Metabolize HEP in Humans

**Figure 3:**
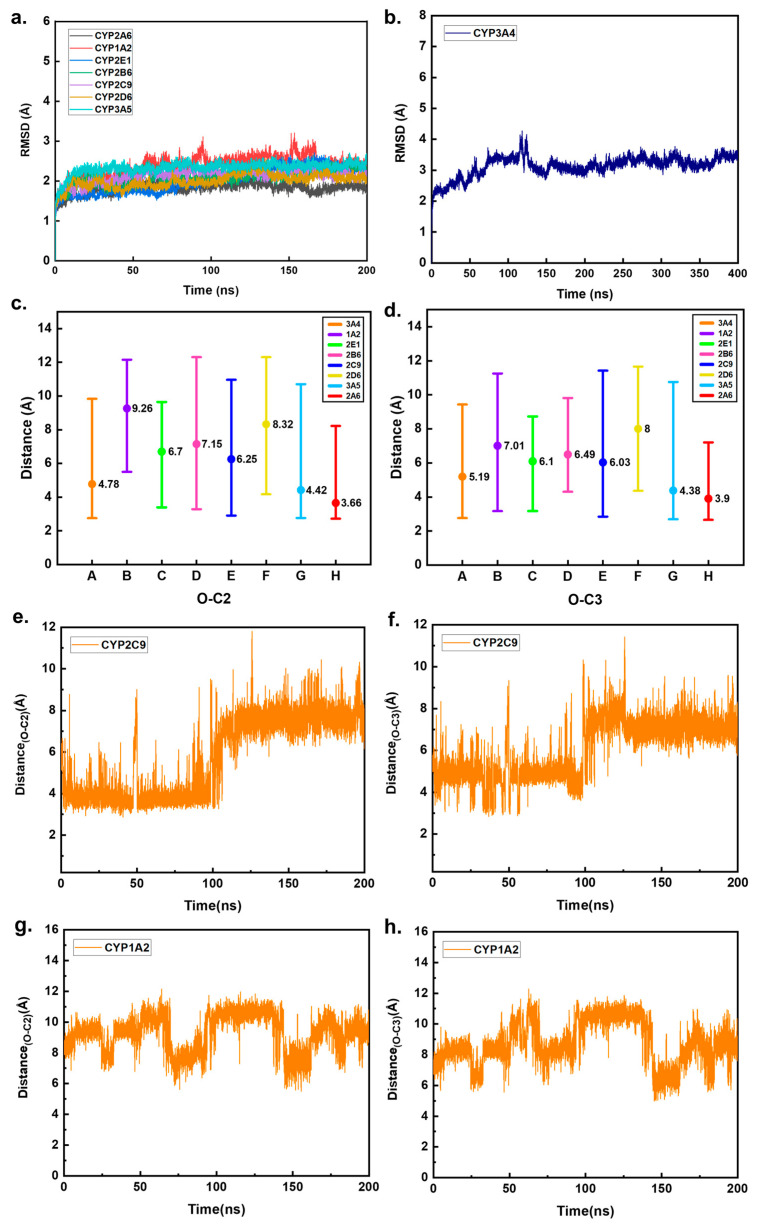
(**a**,**b**) Variation of RMSD with simulation time for eight complex systems. (**c**,**d**) The mean and range of O-Cx distances in eight complexes. (**e**,**f**) The fluctuation of O-Cx distances in the CYP2C9-HEP system. (**g**,**h**) The fluctuation of O-Cx distances in the CYP1A2-HEP system.

MD simulations were performed for 200 ns for each of the eight complexes, and the initially simulated conformations were the optimal complex structures obtained via molecular docking. To assess the stability of the complex structures, the root mean square deviation (RMSD) of each trajectory was calculated. Two sets of parallel simulations for each complex eventually reached equilibrium, and one set was selected from each system for further analysis. The RMSD values are shown in Figure 3a,b. All complexes, except CYP1A2-HEP and CYP3A4-HEP, were stabilized during the last 100 ns of the simulation, indicating that these six complexes reached equilibrium. The RMSD of the CYP1A2-HEP system fluctuated slightly between 150 ns and 175 ns. Upon examining the dynamic trajectory, we found that it was caused by oscillations of the protein’s terminal residues, which were located far from the protein’s active center and did not affect the substrate–protein interaction. The RMSD of the CYP3A4-HEP system did not sufficiently equilibrate within the initial 200 ns to ensure its stability. Therefore, the MD simulation of the system was extended for an additional 200 ns, with the result presented in Figure 3b. After an extended period, the RMSD leveled off, indicating that the complex system had reached equilibrium.

During enzyme catalysis, the spatial binding pattern between the enzyme and the substrate is an essential driver for the selectivity of the enzymatic metabolic reaction [27,28]. The related literature suggests that for the epoxidation reaction of a double bond, the distance between the activated carbon atom (specifically C2 = C3 of HEP, as shown in Figure 1) and the oxygen atom of Cpd I is required to be less than 4.0 Å [27]. Therefore, we measured the distance between the oxygen atom and the C2/C3 of HEP in all snapshots during the simulations of the eight systems. The CYP isoforms that may catalyze the epoxidation of HEP were qualitatively identified based on the proportion of snapshots that met this criterion.

The average, maximum, and minimum values of the O-Cx (x = 2, 3) distances in the simulated trajectories of all complex systems are shown in Figure 3c,d. It can be seen that only in CYP2A6-HEP, CYP3A4-HEP, and CYP3A5-HEP are the average distances of O-Cx around 4 Å. Because of the large active cavity of CYP3A4, HEP exhibited significant inversion, with the O-Cx distances fluctuating significantly during the simulation (Figure S1) [29,30]. The percentages of snapshots with O-Cx distances not exceeding 4 Å in the simulation trajectories of the eight complexes are shown in Table 1. Notably, the proportion of snapshots with O-Cx distances less than 4 Å is 0% in CYP1A2-HEP and CYP2D6-HEP, while it is also extremely low (<1%) in CYP2E1-HEP and CYP2B6-HEP. Therefore, they can be considered outside the range of the criteria. Notably, although the RMSD of the complex system reached equilibrium at around 50 ns of the simulation, the O-Cx distances of CYP2C9-HEP increased significantly during the last 100 ns of the simulation, greatly exceeding 4 Å (Figure 3e,f). This was not the case for the other three complexes with shorter O-Cx distances (Figure S1). Therefore, it can be concluded that the O-Cx distance of CYP2C9-HEP exceeded 4 Å when the system was stabilized, thus eliminating CYP2C9 as a candidate.

### 2.3. Key Factors Affecting the Binding Site of HEP in CYP Enzymes

To further understand the binding of HEP to the eight enzymes during the simulations, we performed binding free-energy calculations and sampled conformational analysis. We selected a 30 ns segment of the conformation that meets the following criteria: the RMSD is in equilibrium, the conformation is stable during this period, and this conformation accounts for the largest proportion among the simulations with stable RMSD to calculate the binding free energy using the MM-GBSA method. The last snapshots of these trajectories were used as sampled conformations to analyze the conformational changes that occurred during the simulations. The total binding free energies are listed in Table 2. The contribution of the van der Waals term to the binding free energy significantly exceeded that of the electrostatic term for all the complexes, suggesting that HEP primarily interacts with proteins through hydrophobic interactions. The residues whose ΔG_bind_ absolute value is greater than 4 kJ/mol in at least one complex are presented in Table 3, corresponding exactly to those that constitute the four binding pockets described above.

The similarity among the eight sampled conformations decreased slightly compared to that of the crystal structures, which indicated that the conformations of the proteins changed somewhat during the simulation because of their inherent flexibility and participation of HEP [31,32]. Differences in the intrinsic hydrophobic pockets of several enzymes, along with conformational changes during simulations, ultimately resulted in differences in the distance between the barycenter of HEP and Cpd I, as well as the different orientations of the double bond. The combination of these two factors led to greater discrepancies in the O-C_X_ distances between the eight complexes.

**Figure 4:**
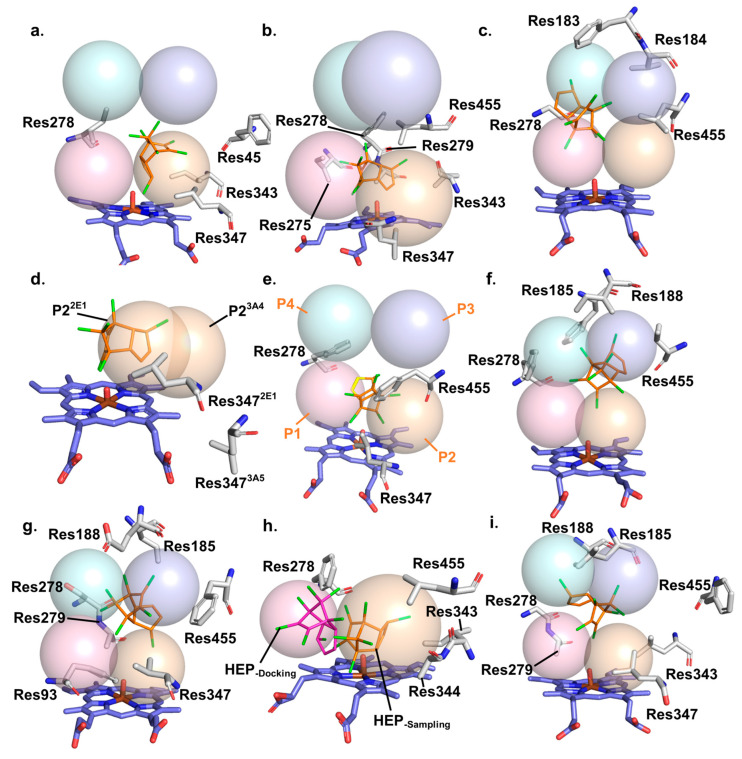
The binding position of HEP in the conformation of the last frame of the simulation trajectory for binding free energy calculation. The enzyme components are represented as bars and HEP is depicted as line. Cpd I (without cysteine) is presented in slate blue and the residues forming the pockets are presented in white in all the pictures. The relative positions of P1, P2, P3, and P4 are shown in (**e**). (**a**) The binding position of HEP in CYP2A6-HEP. (**b**) The binding position of HEP in CYP3A5-HEP. (**c**) The binding position of HEP in CYP1A2-HEP. (**d**) The size difference of the P2 pocket when Res347 and HEP are on the same side of Cpd I and when they are not on the same side of Cpd I. (**e**) The binding position of HEP in CYP2E1-HEP. The yellow part emphasizes the orientation of the double bond in HEP. (**f**) The binding position of HEP in CYP2B6-HEP. (**g**) The binding position of HEP in CYP2D6-HEP. (**h**) Comparison of the docking position of HEP in CYP3A4 and its position in the sampled conformations of CYP3A4-HEP. (**i**) The binding position of HEP in CYP2C9-HEP.

By combining the binding free energy, sampled conformations, and dynamics of the complex during the simulation, the role of each residue in substrate binding could be discussed quantitatively. In CYP2A6-HEP, HEP was initially docked between the P1 and P2 pockets. During the simulation, although Res455 left the binding position of HEP due to random solvent fluctuations, the binding position of HEP in CYP2A6 did not change much compared to the docking position (Figure 4a), which might be attributed to the rigidity of the CYP2A6 active site [33,34]. HEP in CYP3A5-HEP was located at the junction of the P1 and P2 pockets, with its main body positioned within the P2 pocket (Figure 4b). Similarly, its binding position did not change significantly throughout the simulation compared with the docking position. Binding free-energy analysis also showed that all the residues that had strong interactions with HEP belonged to the P1 and P2 pockets.

The docking position of HEP in CYP1A2-HEP is similar to that in CYP3A5-HEP. The main body of HEP was docked in the P1 pocket, whereas a small portion extended into the P4 pocket (Figure 2b). During the simulation, under the influence of Res184 and Res185, which belong to the P4 pocket, HEP moved further toward the P4 pocket and was ultimately bound between the P1 and P4 pockets (Figure 4c). As shown in Figure 3g,h, which are the graphs of O-Cx distance fluctuation in this system, it can be observed that HEP occasionally moved between the P1 and P4 pockets during the simulation process. Per-residue binding free-energy decomposition also showed that the residues exhibiting strong interactions with HEP all belonged to the P1 and P4 pockets. The docking position of HEP in CYP2E1-HEP was slightly closer to the P1 pocket than in CYP3A5-HEP. This may be related to the fact that the Res347 in CYP2E1-HEP is different from that in CYP3A5-HEP, which may be a reason for the minimal CYP2E1 active site [35]. In CY2E1-HEP, Res347 was located on the same side of Cpd I as HEP, which reduced the size of the P2 pocket and further compressed HEP into the P1 pocket. The size of the binding pocket also affected the orientation of C2 = C3 in HEP. The volume of the end containing C2 = C3 was smaller than that of the end containing the chlorine substitution. Moreover, the P1 pocket was narrower than the P2 pocket. When the P2 pocket was large, the HEP tended to enter the P2 pocket more frequently during the simulation, allowing for greater flipping. The flipping motion brought C2 = C3 closer to Cpd I. However, when Res347 compressed the P2 pocket, HEP was inserted deeper into the P1 pocket and failed to flip sufficiently. Instead, it joined to the P1 pocket using its smaller end. The combined effects of the barycenter position and orientation of the double bond caused the double bond of HEP in CYP2E1-HEP to move farther away from Cpd I (Figure 4b,d,e). Residue-binding free-energy decomposition analysis indicated that more residues in CYP2E1-HEP exhibited strong interactions with HEP, which may be attributed to its smaller binding space.

For CYP2B6-HEP, the binding position of HEP during simulation was slightly deeper within the P3 pocket than at its docking position, primarily forming interactions with the residues of this pocket (Figure 4f). Res93 in CYP2D6-HEP is phenylalanine, a large hydrophobic amino acid that differs from the corresponding residues in other enzymes. In the initial docking, this residue was positioned between Cpd I and HEP, resulting in HEP being located far from Cpd I. Res455, pointing to the P3 pocket, also caused HEP to be located far away from Cpd I. During the simulation, the presence of Res185 and Res188 at the bottom of the P3 pocket allowed HEP to penetrate further into the P3 pocket (Figure 4g). Res93, all the residues in the P3 pocket, and some residues in the P2 pocket formed strong interactions with HEP. Previous studies on Res93 targeted mutation in CYP2D6 have also demonstrated the importance of Res93 in substrate binding and catalysis [36,37].

In CYP3A4-HEP, HEP was docked to the deepest part of the P1 pocket. However, Res344 and Res343 in the P2 pocket successively pulled HEP via hydrophobic interactions, causing it to move toward the P2 pocket during the simulation. When HEP is moved to the junction of the P1 and P2 pockets, Res455 can also interact with it (Figure 4h). Ultimately, the binding position of HEP in CYP3A4 was similar to that in CYP3A5. The residues that strongly interacted with HEP were comparable for both enzymes. Previous studies have shown that CYP3A4 and CYP3A5 have similar secondary and tertiary structures. However, HEP was more easily flipped in CYP3A4 (Figure S1). This may be attributed to the distinct shapes of the active cavities. The active cavity of CYP3A5 was narrower and taller than that of CYP3A4, and the size of HEP was better fitted to the width of the cavity of CYP3A5, which made the binding tighter, with HEP less likely to flip [38,39]. Due to the absence of the obstruction of Res278 and Res455, there was more space for HEP to move around in CYP2C9-HEP. Therefore, movement of the HEP and frequent flipping of its orientation were observed during the simulation. Eventually, HEP tended to bind between the P2 and P3 pockets, favoring the P3 side. Most residues that interacted strongly with HEP belonged to the P3 pocket (Figure 4i).

In summary, the binding position of HEP in the enzyme is not a random result, but an inevitable consequence determined by the shape and composition of the hydrophobic pocket near Cpd I. First, Res278 and Res455 act like two doors, dividing the entire binding region into two parts: one closer to Cpd I and the other farther away. The species of these two residues in the different enzymes have a substantial effect on the initial docking positions of HEP. In particular, HEP in CYP2D6-HEP is far from Cpd I, primarily because Res93 is inserted between HEP and Cpd I. Additionally, the binding positions of HEP during simulations are mainly influenced by the shape of the hydrophobic pockets and the key residues that strongly interact with HEP. When the hydrophobic pocket near Cpd I was much larger than the size of the HEP, the HEP tended to turn over during the simulation. Conversely, HEP binds more stably to the enzyme when the binding pocket is closer to that of HEP. When the shape of the binding pocket permits, residues that can have strong hydrophobic interactions with HEP can also influence its binding position.

### 2.4. Potential Energy Surface Analysis of HEP Epoxidation Catalyzed by Different CYP Enzymes

QM calculations were performed to investigate the epoxidation of HEP by the CYP enzymes. The initially linked Cpd I-HEP may exhibit multiple conformations that can be interconverted through a process with a lower potential barrier under free linkage. However, only a limited number of Cpd I-HEP conformations are stabilized in CYP enzymes. We selected several atoms that significantly influenced the relative positional relationship between Cpd I and HEP, including the iron and oxygen atoms in Cpd I and C2, C3, C10, Cl1, and Cl7 in HEP (Figure 1a). The simulated conformations of CYP2A6-HEP, CYP3A4-HEP, and CYP3A5-HEP were clustered based on the RMSD of these atoms. The Cpd I-HEP conformations that take a substantial proportion and have at least one of the O-C2 or O-C3 distances measuring less than 4 Å were extracted to serve as initial reactants for QM calculation. Two conformations were extracted from each of the three complexes and designated as 2A6_1, 2A6_2, 3A4_1, 3A4_2, 3A5_1, and 3A5_2. The proportions of these conformations in the simulated trajectories of the three complexes are listed in Table 4. The proportion of 3A4_2 was only 0.08%, which can be assumed not to be easily formed in CYP3A4-HEP owing to the surrounding enzyme environment. Therefore, it was excluded from the subsequent calculations. The configurations of the six initial reactants are illustrated in Figure S2. As shown in the figure, 3A5_2 and 2A6_1 are very similar in configuration and have the same minimum energy point after optimization using the unrestricted B3LYP functional. Therefore, they can be considered the same initial reactants and possess the same epoxidation potential energy surface. The same was true for 3A4_1 and 3A5_1. In the potential energy surface diagrams and tables, 3A5_2 and 2A6_1 are referred to as 3A5_2, and 3A4_1 and 3A5_1 are referred to as 3A4_1. Furthermore, 2A6_2 still uses its name.

**Scheme 1:**
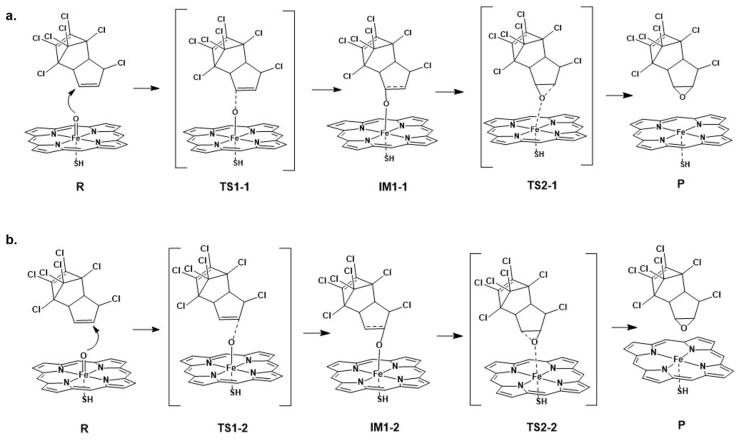
The proposed two mechanisms of HEP metabolism in CYPs. (**a**) The epoxidation reaction route in which O attacks C3 first. (**b**) The epoxidation reaction route in which O attacks C2 first.

Next, the potential energy surfaces for the epoxidation of HEP were calculated using each of the three initial reactants. The reaction schemes for the epoxidation pathway are shown in Scheme 1a,b, where the oxygen atom initially attacks one of the carbon atoms in the double bond at random. In the practical calculations, we determined the initial attack sites of the oxygen atom in different configurations according to the proximity principle. In 3A5_2 and 2A6_2, C3 was attacked first, whereas in 3A4_1, C2 was attacked first. The energy distributions of the epoxidation pathways and the proportions of the initial reaction conformations in the simulated trajectories are presented in Table 4. In contrast to 3A5_2 and 3A4_1, which proceed stepwise, the epoxide was formed directly from TS1-3 in the epoxidation pathway of 2A6_2. This is consistent with de Visser’s conclusion that the epoxidation processes of alkenes catalyzed by the doublet state of Cpd I are effectively concerted [40,41,42]. Also, 3A5_2 has an extremely low ring closure barrier. From the results of the single-point energy calculations for the 3A4_1 reaction pathway, it appears that the energy of TS2-2 is slightly lower than that of IM1-2. When we performed IRC calculations at the theoretical level of structure optimization, TS2-2 was connected to IM1-2 and P2 as the highest point, indicating that the obtained transition state structure was accurate. This may be because a higher-level basis set was used when calculating the single-point energy. The fact that the energy of the transition state is higher than that of the intermediates is guaranteed to hold only at the theoretical level of structural optimization [43,44,45,46]. This also indicates that the energy of TS2-2 was only slightly higher than that of IM1-2. These results confirm that the epoxidation pathway of HEP catalyzed by a low-spin double-state Cpd I is nonsynchronous but effectively concerted.

**Figure 5:**
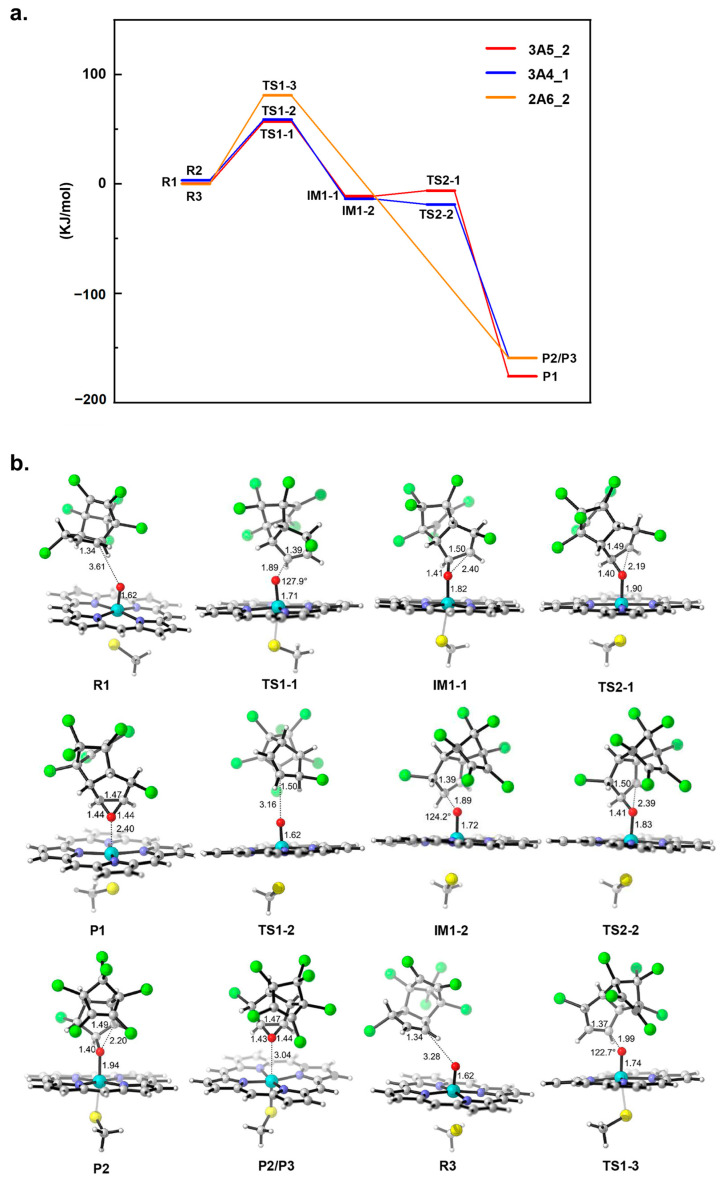
(**a**) Energy profiles of the three metabolic pathways. (**b**) Optimized structures of reactant (R), transition state (TS), intermediate (IM), and product (P). C, O, Cl, N, Fe, S and H are represented by gray, red, green, purple, blue, yellow and white respectively.

The relative energies of the reactants, intermediates, transition states, and products are shown in Figure 5a, and their corresponding structures are shown in Figure 5b. The three initial conformations along the epoxidation pathway exhibited consistent trends in their geometric conformational changes. Initially, the oxygen atom of Cpd I performed an electrophilic attack on either C2 or C3 to reach transition state TS1. During this process, both the Fe-O bond and C2=C3 bonds lengthened. The geometries of TS1-1, TS1-2, and TS1-3 are all close to the ideal TS geometries calculated by Coleman et al. The C2/C3-O distance is approximately 1.98 Å, and the Fe-O-C2/C3 angle is around 127° [47]. The changes in geometric parameters from R to IM1 in the pathways of 3A4_1 and 3A5_1 are also similar. During the transition from TS1 to IM1, the Fe-O bond length increased by 0.11 Å, and the C2-C3 bond length increased by 0.11 Å. The distance from the oxygen atom to the other carbon atom in the double bond decreases during the transition from IM1 to TS2, and the Fe-O bond continues to lengthen. From TS2 to P, the oxygen atom forms a bond with another carbon atom. The lengths of the two O-C bonds in P1 and P2/P3 were essentially equal. However, P1 and P2/P3 did not share the same energy minimum, and their configurations were different. P1 is the exo-epoxide, while P2/P3 are the endo-epoxides. The counterpart to this is that in 3A4-1 and 2A6-2, the oxygen atom attacks the carbon atom from the same side of the HEP, whereas in 3A5_2, it attacks the carbon atom from the opposite side. According to de Visser’s study, the epoxidation intermediates of alkene epoxidation catalyzed by the doublet state of Cpd I are ultrashort-lived and produce epoxide products instantaneously without conformational change due to rearrangement during this process [40,41,42]. Consequently, it can be considered that the configurations of products depend on the initial attacked orientation of the oxygen atom during the epoxidation process of HEP.

The energy span from R to TS1 was the largest for all three potential energy surfaces, indicating that the electrophilic attack of the oxygen atom on C2 or C3 was the rate-controlling step of the entire epoxidation reaction. The energy barriers for the rate-controlling steps of 3A5_2 and 3A4_1 were very similar, measuring 56.76 kJ/mol and 55.68 kJ/mol, respectively. In contrast, the energy barrier for the rate-controlling step of 2A6_2 epoxidation was 81.06 kJ/mol. These differences in potentials confirm the significant influence of the initial conformation of the enzyme–substrate complex on the oxidizing activity [48,49,50]. The energy barrier for the rate-controlling step of 2A6-2 epoxidation is higher than that of 3A5_2 and 3A4_1, indicating that the reaction with 3A5_2 and 3A4_1 as the initial configurations is thermodynamically more favorable than the reaction with 2A6-2 as the initial configuration. Combined with their respective proportions in the simulated trajectories of the CYP2A6-HEP, CYP3A4-HEP, and CYP3A5-HEP systems, we screened for CYP isoforms that may metabolize HEP in humans. During the simulation of the CYP2A6-HEP system, the O-Cx distances in 59.55% of the conformations fell within the range conducive to epoxidation and 33.78% were in the reaction pathway with a lower rate-controlling step barrier. In the simulation of the CYP3A4-HEP system, 32.80% of the conformations were in the reaction pathway with a lower rate-controlling step barrier. For the CYP3A5-HEP system, both 3A5_1 and 3A5_2 were in the reaction pathway with a lower rate-controlling step barrier, together accounting for 69.81% of the simulated conformations. Therefore, among these three enzymes, CYP3A5 is the most likely candidate enzyme to catalyze the epoxidation of HEP in the human body. CYP3A4 and CYP2A6 may also metabolize heptachlor, but their activities are lower than that of CYP3A5. Numerous experiments in the past have proven that both CYP3A4 and CYP3A5 have a very wide range of substrate types. They exhibit significant overlap in substrate specificity but sometimes differ in product regioselectivity and formation activity [51,52,53,54]. CYP2A6 has been proven to be a key factor in the metabolic activation of many chemical carcinogens. Its classic metabolic substrates, such as coumarin, steroids, and nicotine, are all small molecules with planar rings, similar to heptachlor [55,56,57,58].

## 3. Materials and Methods

### 3.1. Model Preparation

We selected eight human CYP enzymes based on their abundance in the human liver and their involvement in the metabolism of exogenous substances in vivo [59,60]. The selected enzymes were CYP1A2, CYP2A6, CYP2B6, CYP2C9, CYP2D6, CYP2E1, CYP3A4, and CYP3A5. Six of these CYP isoforms, including CYP1A2 (PDB ID: 2HI4), CYP2A6 (PDB ID: 1Z10), CYP2B6 (PDB ID: 3IBD), CYP2C9 (PDB ID: 4NZ2), CYP2D6 (PDB ID: 5TFT), and CYP3A5 (PDB ID: 5VEU), have complete crystal structures available in the Protein Data Bank (http://www.rcsb.org, accessed on 5 March 2023) [39,61,62,63,64,65,66,67]. These crystal structures, after the removal of the original ligand, can be used for subsequent studies. However, currently available crystal structures of CYP2E1 and CYP3A4 are incomplete. To obtain the complete crystal structure of CYP2E1, we selected the crystal structure of CYP2E1 (PDB ID: 3E6I) with one missing residue and used the Model Loops panel in Chimera to add the missing residue [35,68]. The existing crystal structures of CYP3A4 all lack the N-terminal helix. Therefore, we selected the crystal structure of CYP51 (PDB ID:4LXJ), which has a complete N-terminal helix, along with CYP3A4 (PDB ID:1TQN) as templates to obtain the complete crystal structure of CYP3A4 using the multi-template modeling module of Modeller 9.23 [29,69,70,71]. Finally, we manually added an oxygen atom to the iron atom of the heme by GaussView 6.0 to construct the active iron (IV)-oxo porphyrin species, Compound I (Cpd I), in each protein [72,73]. The initial 3D structure of HEP (CID: 3589) was obtained from the PubChem database (https://pubchem.ncbi.nlm.nih.gov, accessed on 6 March 2023) and was optimized at the B3LYP/6-31G (d, p) level by Gaussian 16 program [74,75,76,77,78,79,80].

### 3.2. Molecular Docking and Molecular Dynamics Simulation

The protonation states of the titratable residues (pH 7.4) were determined using the H++ Web server (http://biophysics.cs.vt.edu/H++, accessed on 10 March 2023) [81,82,83]. The optimized HEP was docked five times with each CYP isoform using AutoDock 4.2.6, with different sizes of grid boxes selected for each docking, for a total of 150 docked conformations for each CYP isoform [84]. The structures of these eight proteins were all obtained by the X-ray diffraction method. In the docking process, the crystallographic coordinate system of the original PDB structure was adopted, with the origin of the original crystallographic coordinate system serving as the origin. The docking was carried out with the centroid of the active center as the grid center. The coordinates of the grid centers for the eight enzymes are as follows: x = 7.31, y = 21.57 and z = 25.26 (CYP1A2), x = 72.46, y = 77.27 and z = 102.93 (CYP2A6),x = 20.49, y = 8.99 and z = 20.46 (CYP2B6), x = −55.37, y = −48.85 and z = −20.73 (CYP2C9), x = −7.79, y = 26.47 and z = −3.95 (CYP2D6), x = 30.25, y = 33.68 and z = 20.24 (CYP2E1), x = −20.35, y = −24.77 and z = −15.21 (CYP3A4), x = −37.36, y = −0.33 and z = 22.86 (CYP3A5). The size of the grid boxes was 40.0 × 40.0 × 40.0 Å^3^, 45.0 × 45.0 × 45.0 Å^3^, 50.0 × 50.0 × 50.0 Å^3^, 55.0 × 55.0 × 55.0 Å^3^, and 60.0 × 60.0 × 60.0 Å^3^. The optimal complex conformations were screened based on both the binding energy of the complexes and the docking positions of HEP.

Force field parameters were assigned to the complexes using the tleap module of the AmberTools 16 [85]. The force field of protein was provided by ff14SB, and the force field parameters for Cpd I were obtained from the work of Shalrokh et al. [86,87]. The Antechamber module with the Generalized Amber force field (GAFF) was used to generate the force field parameters of HEP [88,89]. To account for the solvent environment, the systems were solvated with the TIP3P water box with 12 Å between the solute boundary [90]. Each system was kept electrically neutral by the addition of a corresponding number of counterions.

All trajectories were performed using the AMBER 16 software package [85]. To reduce undesirable interatomic contacts, each system was first minimized by 20,000 steps of the steepest descent minimization and 10,000 steps of the conjugate gradient minimization under a limiting potential constraint of 8.37 × 10^−2^ kJ mol^−1^nm^−2^. Then, the same steps of energy minimization without any constraint were performed repeatedly. Next, the systems were gradually heated from 0 K to 310 K within 50 ps, with a force constraint of 4.19 × 10^−2^ kJ mol^−1^Å^−2^ in each protein, Cpd I, and HEP in the NVT ensemble. Subsequently, 1.5 ns equilibrium simulations were performed for the systems in the NPT ensemble. Finally, 200 ns MD simulations were performed for the complex systems in the NPT ensemble. During the MD simulations, the temperature was maintained at 310 K by Langevin dynamics [91]. A constant isotropic pressure was maintained at the atmospheric pressure through a Berendsen barostat approach [92]. In addition, the cut-off value for short-range interactions was set as 1.0 nm, and the long-range electrostatic interaction was handled by applying the Particle Mesh Ewald (PME) method [93]. All covalent bonds containing hydrogen atoms were dealt with by the SHAKE algorithm [94]. The time step was set to 2 fs. Data analysis was performed using the cpptraj module of AmberTools 16 [85]. Two sets of parallel simulations were performed for each system to eliminate randomness of the results.

### 3.3. Binding Free-Energy Calculation

In the simulation of each complex system, a 30 ns MD simulation trajectory with a relatively stable RMSD was selected to perform the binding free-energy calculation using the Molecular Mechanics Generalized Born Surface Area (MM/GBSA) method [95]. The complex binding free energy (ΔG_bind_) is defined as follows:

ΔG_bind_ = ⟨G_complex_⟩ − ⟨G_CYP_⟩ − ⟨G_HEP_⟩ (1)

G_complex_, G_CYP_, and G_HEP_ are the free energies of the complex, CYP, and HEP, respectively. The free energy can be calculated as follows:

G_x_ = ⟨E_MM_⟩ + ⟨G_solv_⟩ − TS (2)

E_MM_ = E_ele_ + E_vdw_ + E_int_ (3)

G_solv_ = G_GB_ + G_SA_ (4)

E_MM_ represents the component of molecular mechanisms in the gas phase, which consists of the electrostatic term (E_ele_), the van der Waals interaction term (E_vdW_), and the internal interaction term (E_int_). G_solv_ represents the contribution of stabilization energy, consisting of the polar term (G_GB_) and the nonpolar term (G_SA_). G_solv_ is the free energy of electrostatic solvation, consisting of a polar term (G_GB_) and a nonpolar term (G_SA_). TS represents the contribution of entropy, and the contribution of gas-phase entropy can be obtained by the regular pattern analysis method. However, this calculation is expensive and lacks high reproducibility, with large errors. The differences in the number of residues among the CYP isoforms studied in this paper are minimal, and the secondary structure is highly conserved, so we neglected the entropy contribution when analyzing the binding free energy.

### 3.4. Quantum Mechanical Calculation

In the CYP-catalyzed epoxidation reaction, the main catalytic function is performed by Cpd I [73]. Residues surrounding the binding pocket do not participate in catalysis but mainly serve to locate the substrate. Consequently, we selected only HEP, Cpd I (simplified to retain only the porphyrin ring and Fe=O), and cysteine, which immobilizes Cpd I for the QM calculation region. To consider the effect of the localization of the surrounding residues, we performed cluster analysis based on the positional relationship between HEP and Cpd I. The regions used for the QM calculation were extracted from the conformations with the highest probability of reaction and the largest proportions [85]. These conformations were utilized as the initial reactants for the QM calculation. Notably, less proportional conformations are less likely to form because of the constraints imposed by the shapes of the binding pockets. Therefore, they exert less influence on the subsequent catalytic reactions. We then calculated the potential energy surfaces of the epoxidation reaction for all initial reactants separately by the Gaussian 16 [76]. All calculations employed the unrestricted B3LYP functional [80]. Geometric optimizations and frequency calculation of the reactants, transition states (TS), intermediates (IM), and products in the reaction system were performed using 6–31 G (d, p) (C, H, O, N, S) and LANL2DZ (Fe) base sets [96]. The vibrational frequency calculation confirmed that each TS has only one imaginary frequency. Intrinsic reaction coordinate (IRC) calculations were also performed at the same level to validate the reasonableness of the TS structure [97,98,99,100]. Finally, single-point energy calculations, including D3 empirical dispersion corrections and BJ damping for the optimized configurations, were performed using the base set Def2TZVP [101,102,103]. Previous studies have shown that alkenes epoxidation catalyzed by the doublet state of Cpd I has a shorter reaction pathway and is less prone to by-products than that catalyzed by the quartet state of Cpd I [40,41,42,104]. Therefore, we considered only the doublet state of Cpd I in the QM calculations.

## 4. Contributions and Future Plans

This study puts forward new ideas for the innovative application of theoretical simulation methods. It uses a combined approach of MD simulations and QM calculations to replace the cumbersome enzyme phenotype experiments to screen the CYP enzyme subtypes most likely to metabolize HEP in the human body. Meanwhile, it comprehensively explored the metabolic mechanism of HEP in enzymes, providing important guidance for designing inhibitors of HEP epoxidation and for reducing HEP residues in the human body. The main parts of the three-dimensional molecular structures adopted in this study are obtained from experiments. MD calculations can obtain the time-evolution process of various microscopic particles in the system. They have been widely applied to the study of protein–ligand interactions and the method of judging metabolic specificity by the distance between the ligand site, and the enzyme active center during the simulation process has also been used in many studies [105,106,107,108]. Many studies have also employed QM calculations to explore the reaction mechanisms of molecules in enzymes, and the calculation accuracy of this approach is widely acknowledged [73,109,110,111,112]. Therefore, the theoretical calculation methods adopted in this study are highly reliable, and the obtained results are consistent with the proven metabolic characteristics of enzymes.

Since the idea of using theoretical calculation methods to replace enzyme phenotype experiments has not been systematically verified before, systematic experimental verification is still needed for this method to be widely applied in the future. Therefore, in the subsequent research, we will cooperate with research groups conducting relevant experiments. By incubating the recombinant CYP enzymes with HEP in the NADPH regeneration system and analyzing the products using the Liquid Chromatography-Tandem Mass Spectrometry (LC-MS/MS) method, we will experimentally study the metabolism of HEP by the eight CYP subtypes mentioned in this paper to verify the theoretical calculation results and provide an experimental basis for the new application of the theoretical simulation method proposed in this paper. In addition, HEPX exists in two configurations that are capable of interconversion. After a prolonged period in the human body, it can undergo further metabolism. Currently, the conversion conditions between the two configurations, as well as the metabolic cycle and process, remain unclear. In the future, once an appropriate opportunity arises, we plan to conduct targeted research, striving to fill the knowledge gap in this area and provide a scientific basis for a deeper understanding of the metabolism of heptachlor in the human body.

## 5. Conclusions

This study provides a comprehensive description of the binding modes and metabolic pathways of HEP in human CYPs at the microscopic level and screens the CYP enzyme most likely to metabolize HEP in humans by molecular docking, MD simulations, and QM calculations. The results showed that HEP mainly binds to hydrophobic pockets near the active sites of the enzymes. The different compositions and shapes of these hydrophobic pockets result in differences in the binding positions and postures of the HEP in different enzymes. Only in CYP2A6, CYP3A4, and CYP3A5 did the distance from the oxygen atom of Cpd I to the reaction site on HEP meet the criteria for performing the epoxidation reaction during simulations. Three initial reaction structures were extracted from three complex simulation trajectories for QM calculations. The epoxidation pathways of HEP catalyzed by low-spin double-state Cpd I are nonsynchronous but effectively concerted. The rate-controlling step in these epoxidation pathways is electrophilic attack. The energy barriers of the rate-controlling steps differed among the three initial reactants. Considering both the energy barriers of the rate-controlling step and the clustering ratios, it can be concluded that CYP3A5 is the most likely candidate for catalyzing HEP epoxidation.

## Figures and Tables

**Table 1:** Percentage of snapshots with O-C_X_ distances less than 4 Å in simulation trajectories of eight complexes.

CYPs	O-C2	O-C3	At Least One
CYP1A2	0%	0%	0%
CYP2A6	82.43%	58.31%	92.47%
CYP2B6	0.74%	0.01%	0.75%
CYP2C9	27.4%	8.26%	27.78%
CYP2D6	0%	0%	0%
CYP2E1	0.01%	0.94%	0.94%
CYP3A4	42.74%	9.58%	46.99%
CYP3A5	39.21%	30.32%	63.36%

**Table 2:** Binding free energies between CYPs and HEP (KJ/mol).

Energy Contribution	CYP2B6-HEP	CYP3A5-HEP	CYP2A6-HEP	CYP2E1-HEP	CYP3A4-HEP	CYP1A2-HEP	CYP2D6-HEP	CYP2C9-HEP
ΔE_vdW_	−137.76	−137.18	−153.59	−168.57	−133.03	−142.24	−140.40	−129.05
ΔE_elec_	−22.40	−0.25	3.10	−12.14	−5.11	−3.60	−5.27	−11.51
ΔG_GB_	49.81	21.18	23.89	30.73	31.48	29.34	36.50	35.29
ΔG_SA_	−15.24	−12.85	−15.56	−17.04	−12.85	−15.36	−15.45	−13.69
ΔG_binding_	−137.76	−137.18	−142.17	−168.57	−133.03	−142.24	−140.40	−129.05

**Table 3:** The contributions of significant residues to the binding free energy in eight complexes (KJ/mol).

Pocket	Residues	CYP2B6-HEP	CYP3A5-HEP	CYP2A6-HEP	CYP2E1-HEP	CYP3A4-HEP	CYP1A2-HEP	CYP2D6-HEP	CYP2C9-HEP
P1/P4	Res278	−4.31	−3.51	−6.07	−7.53	−6.15	−2.43	−1.30	−1.13
P1	Res93	−3.43	−1.72	−3.18	−4.69	−1.26	−2.64	−5.56	−2.34
	Res275	−0.29	−4.23	−2.93	−0.02	−1.72	−2.80	−0.84	−0.13
	Res279	−1.63	−3.35	−2.64	−4.52	−5.82	−5.15	−5.98	−5.48
P1/P2	Res283	−1.09	−1.55	−2.30	−5.44	−1.72	−1.38	−1.30	−2.01
P2	Res343	−5.52	−3.10	−5.06	−5.82	−2.26	−0.79	−0.21	−4.81
	Res347	−3.39	−0.33	−5.19	−3.47	−0.42	−2.13	−5.19	−4.14
P2/P3	Res455	−5.61	−4.90	−1.38	−4.23	−4.99	−4.94	−6.15	−0.75
P3	Res188	−5.06	0.03	−3.64	−1.34	−0.08	−1.30	−5.06	−5.69
P3/P4	Res185	−6.78	−3.18	−4.69	−6.19	−0.13	−4.60	−4.10	−8.12
P4	Res184	0.00	−0.96	−1.59	−0.54	−0.08	−4.31	−2.26	−2.13

**Table 4:** Proportions of the three initial reactants in the simulated conformations of the three systems and epoxidation potential energy surfaces (kJ/mol).

	R	TS1	IM2	TS2	P
2A6_1 (33.78%)	0.33	57.10	−11.32	−6.09	−175.98
3A5_2 (32.80%)					
3A4_1 (32.80%)	3.31	58.99	−13.62	−18.77	−159.29
3A5_1 (37.01%)					
2A6_2 (25.77%)	0	81.06	____	____	−159.29

## Data Availability

The raw data supporting the conclusions of this article will be made available by the authors on request.

## Supplementary Materials

The following supporting information can be downloaded at: https://www.mdpi.com/article/10.3390/ijms26052021/s1.
