# Supplementary material for: Theoretical Study on the Metabolic Mechanism of Heptachlor in Human Cytochrome P450 Enzymes
Source: Int J Mol Sci. 2025 Feb 26;26(5):2021. doi: 10.3390/ijms26052021 (PMC11900268; doi:10.3390/ijms26052021)
Supplement: Supplementary file 1 [file ijms-26-02021-s001.zip › ijms-3449550-supplementary.pdf]

Supporting Information for

Theoretical study on the metabolic mechanism of heptachlor in human cytochrome P450 enzymes

Xuerui Zhao[a], Hao Zhang[a], Xiaoli Shen[a], Qingchuan Zheng[b], Song Wang[a]*

a. Institute of Theoretical Chemistry, Jilin University, Changchun, Jilin 130021, China
b. School of Pharmaceutical Sciences, Jilin University, Changchun, Jilin 130021, China

The Supporting Information contains 4 pages, 1 table and 2 figures.

Content of this file

Table S1 Comparison of residues at similar positions in the binding pocket for the eight CYP isoforms.
Figure. S1 The fluctuation of O-Cx distances in CYP2A6-HEP, CYP3A4-HEP and CYP3A5-HEP during simulations.
Figure. S2 the configurations of 2A6_1, 3A4_2, 3A5_2, 3A4_1, 3A5_1, 2A6_2.

Table S1. Comparison of residues at similar positions in the binding pocket for the eight CYP isoforms.

Pocket	CYPs	2B6-HEP	3A5-HEP	2A6-HEP	2E1-HEP	3A4-HEP	1A2-HEP	2D6-HEP	2C9-HEP
P1/P4	Res278	PHE 270	PHE 278	ILE 270	PHE 267	PHE 283	GLY 283	SER 274	GLY 270
P1	Res93	ILE 87	SER 93	VAL 87	ILE 84	SER 98	THR 91	PHE 90	ILE 87
	Res275	LEU 268	ILE 275	ASN 267	ASP 264	ILE 280	ASP 280	ASP 271	ASP 267
	Res279	ALA 271	ALA 279	GLY 271	ALA 268	ALA 284	ALA 284	ALA 275	ALA 271
P1/P2	Res283	THR 275	THR 283	THR 275	THR 272	THR 288	THR 288	THR 279	THR 275
P2	Res343	LEU 335	VAL 343	VAL 335	LEU 332	ILE 348	PHE 348	ILE 339	LEU 335
	Res347	VAL 340	LEU 347	LEU 340	LEU 337	LEU 352	ILE 353	VAL 344	LEU 340
P2/P3	Res455	VAL 450	LEU 455	PHE 450	PHE 447	LEU 461	LEU 464	PHE 453	PHE 450
P3	Res188	ILE 182	GLY 188	THR 182	LEU 179	ASP 193	VAL 197	GLU 186	LEU 182
P3/P4	Res185	ILE 179	LEU 185	PHE 179	PHE 176	LEU 190	VAL 194	LEU 183	ILE 179
P4	Res184	LEU 181	PHE 184	ILE 178	ASN 175	LEU 189	PHE 193	GLY 182	ASN 178

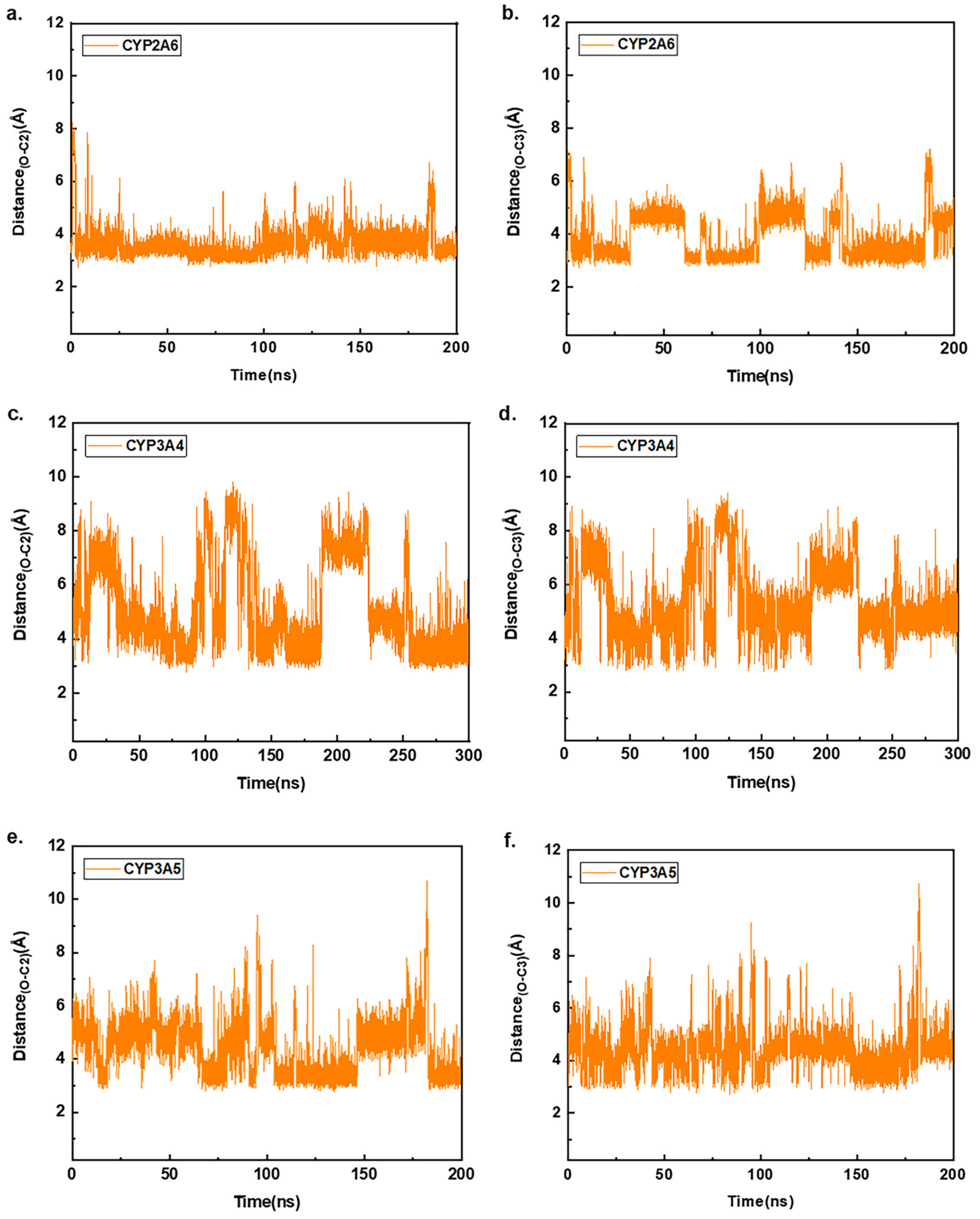

Figure S1. The fluctuation of O-Cx distances during simulations. (a,b) The fluctuation of O-Cx distances in CYP2A6-HEP; (c,d) The fluctuation of O-Cx distances in CYP3A4-HEP; (e,f) The fluctuation of O-Cx distances in CYP3A5-HEP.

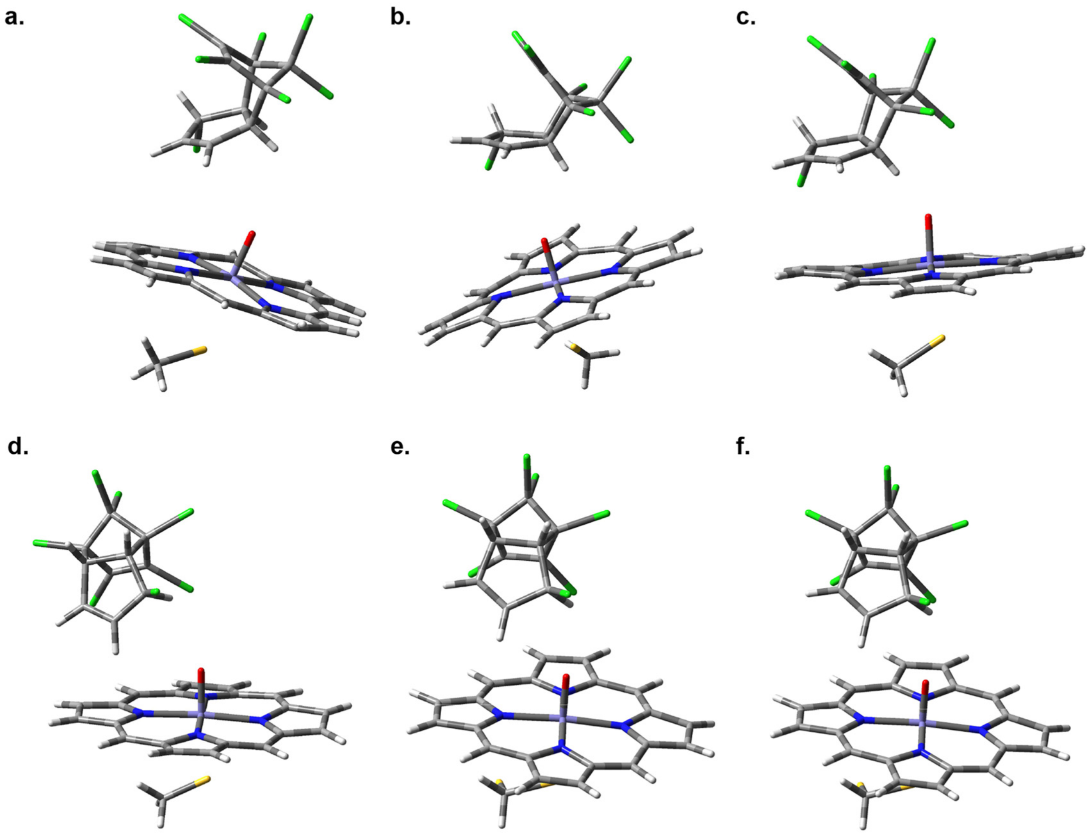

Figure S2. The initial optimal CpdI-HEP configurations. (a) The initial optimal configuration of 2A6_1; (b) The initial optimal configuration of 3A4_2; (c) The initial optimal configuration of 3A5_2; (d) The initial optimal configuration of 3A4_1; (e) The initial optimal configuration of 3A5_1; (f) The initial optimal configuration of 2A6_2.
